# Atrial fibrillation induced by gabapentin: a case report

**DOI:** 10.1186/s13256-023-03975-1

**Published:** 2023-06-09

**Authors:** Sung Hwan Park, Kristen Hunter, Hugh Berry, Yuri Chaves Martins

**Affiliations:** 1 Department of Anesthesiology, Saint Louis University School of Medicine, Drummond Hall-2Nd Floor, 3691 Rutger St., Saint Louis, MO 63110 USA; 2 SSM Health St. Mary’s Hospital, St. Louis, MO USA

**Keywords:** Gabapentin, Atrial fibrillation, Adverse side effects, Case report

## Abstract

**Background:**

Gabapentin is commonly prescribed for the treatment of neuropathic pain, restless leg syndrome, and partial-onset seizures. Although the most frequent side effects of gabapentin are associated with the central nervous system, gabapentin can also affect the cardiovascular system. Case reports and observational studies have showed that gabapentin can be associated with increased risk of atrial fibrillation. However, all the evidence is concentrated in patients older than 65 years old with comorbidities that predispose them to the development of arrhythmias.

**Case presentation:**

We describe a case of an African American male in his 20s that presented to our chronic pain clinic with lumbar radiculitis and developed atrial fibrillation 4 days after being started on gabapentin. Laboratory workup did not show significant abnormalities, including normal complete blood count, comprehensive metabolic panel, toxicology screen, and thyroid-stimulating hormone. Transthoracic and transesophageal echocardiography showed a patent foramen ovale with right-to-left shunt. The patient was initially treated with diltiazem for heart rate control and apixaban. Direct current cardioversion with successful conversion to sinus rhythm was performed 24 hours after admission. The patient was then discharged on apixaban and diltiazem. Apixaban was changed to low-dose aspirin 1 month after discharge.

**Conclusion:**

With rapidly increasing usage of gabapentin for approved and off-label indications, it is important to identify unintended adverse effects of this drug as they are considered safe alternatives to opioids. New-onset atrial fibrillation could be induced by gabapentin in young individuals.

## Background

Gabapentin is used for multiple approved and off-label indications including radicular pain, postherpetic neuralgia, focal onset seizures, restless leg syndrome, and fibromyalgia (1). Gabapentin use and misuse has widely increased recently due to multiple factors, for instance, its prescription as an alternative to opioids to treat chronic pain (1, 2, 3). The most frequent side effects of gabapentin are associated with the central nervous system, such as somnolence and dizziness. Additionally, gabapentin, to a much lesser extent, can also have cardiovascular side effects such as peripheral edema, hypertension, and cardiomyopathy (4, 5).

Atrial fibrillation (AFib) is the most common sustained cardiac arrhythmia (6). Although the prevalence of AFib is high in the elderly (> 10% in persons aged 65 years or older), it is relatively rare in young adults (0.12–0.16% in individuals younger than 49 years) (7). One observational study showed that gabapentin can be associated with increased risk of atrial fibrillation (5). However, all the described evidence was observed in patients older than 65 years old with comorbidities that predispose to the development of arrhythmias.

To the best of our knowledge, no previous case report has shown induction of new-onset Afib by gabapentin in an individual younger than 40 years old. This case report describes an otherwise healthy male in his 20s who presented to our chronic pain clinic with lumbar radiculitis and developed atrial fibrillation 4 days after being started on gabapentin.

## Case description

A previously healthy African American male in his 20s with no known drug allergies or smoking history presented to the chronic pain management clinic complaining of neck and low back pain for 3 months, which started following a motor vehicle collision. He was the driver, seat-belted, going around 75 km/hour when he was hit on the passenger side. The airbag was not deployed, and he did not lose consciousness. The pain was initially located over bilateral neck and lower back. However, 2 months after the accident the pain also started to radiate to bilateral lower extremities in an L5 distribution. The pain was continuous, described as sharp and dull, and was rated as 6.5 out of 10 on a 10-point visual analog scale. Running and extension of the neck were described as worsening factors. Previous evaluations by the emergency department, patient’s primary care physician, and orthopedic spine surgery clinic (on the day of, 5 days and 2 months after the accident, respectively), revealed myofascial pain syndrome. Prior to evaluation at our chronic pain clinic, the patient was prescribed cyclobenzaprine 10 mg per oral every night at bedtime and acetaminophen 650 mg per oral every 6 hours as needed, with minimal relief of the pain. He also was referred to physical therapy, which provided mild temporary relief. Physical examination revealed positive straight leg rise test bilaterally and positive lumbar facet loading tests bilaterally. Lumbar spine X-rays were normal. Cervical spine X-rays showed mild straightening of the cervical spine. Lumbar spine magnetic resonance imaging (MRI) results showed mild disc bulging at L4/L5, small central disc protrusion at L5/S1 level without significant stenosis, and mild bilateral neural foraminal stenosis at L5/S1 level. The patient was initially treated with a fluoroscopically guided caudal epidural corticosteroid injection using 10 mg of dexamethasone, which provided more than 90% pain relief for 1 month. Because relief was significant but short-lived, the patient returned for a bilateral L5 transforaminal epidural corticosteroid injection of 10 mg of dexamethasone, which provided the patient with 50% relief of his pain for 1 month. Due to the short-term pain relief provided by the second procedure, the patient was started on gabapentin 300 mg per oral twice per day and cyclobenzaprine was stopped.

**Fig. 1:**
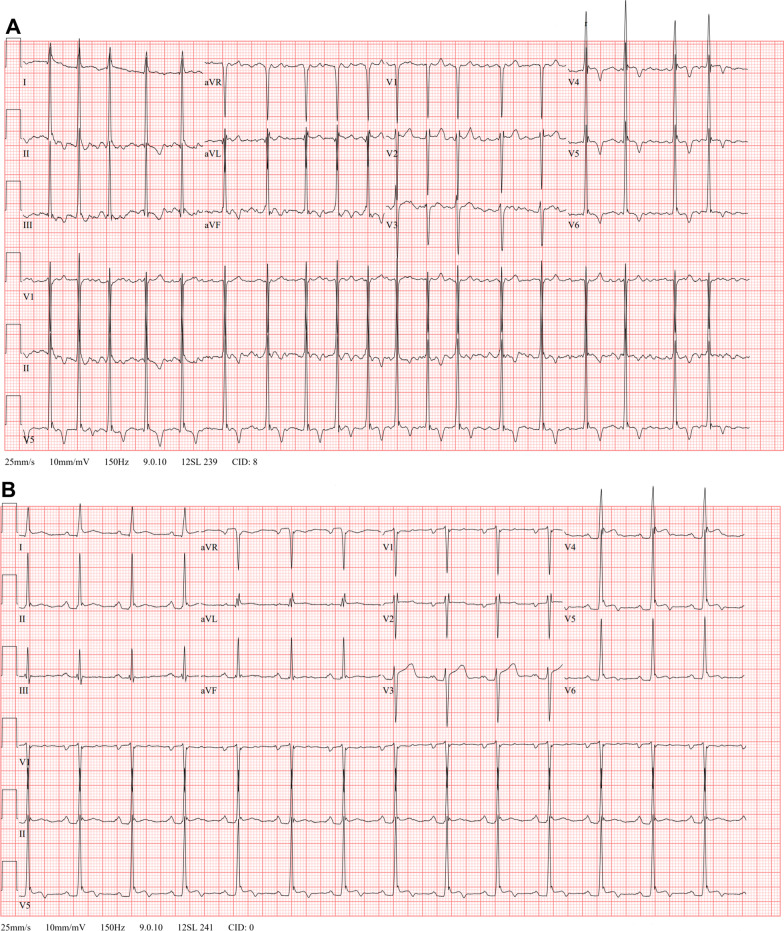
Electrocardiogram at presentation to the emergency department (**A**) and after direct current cardioversion (**B**). Patient meets criteria for left ventricular hypertrophy by the Sokolow–Lyon voltage product criteria

**Fig. 2:**
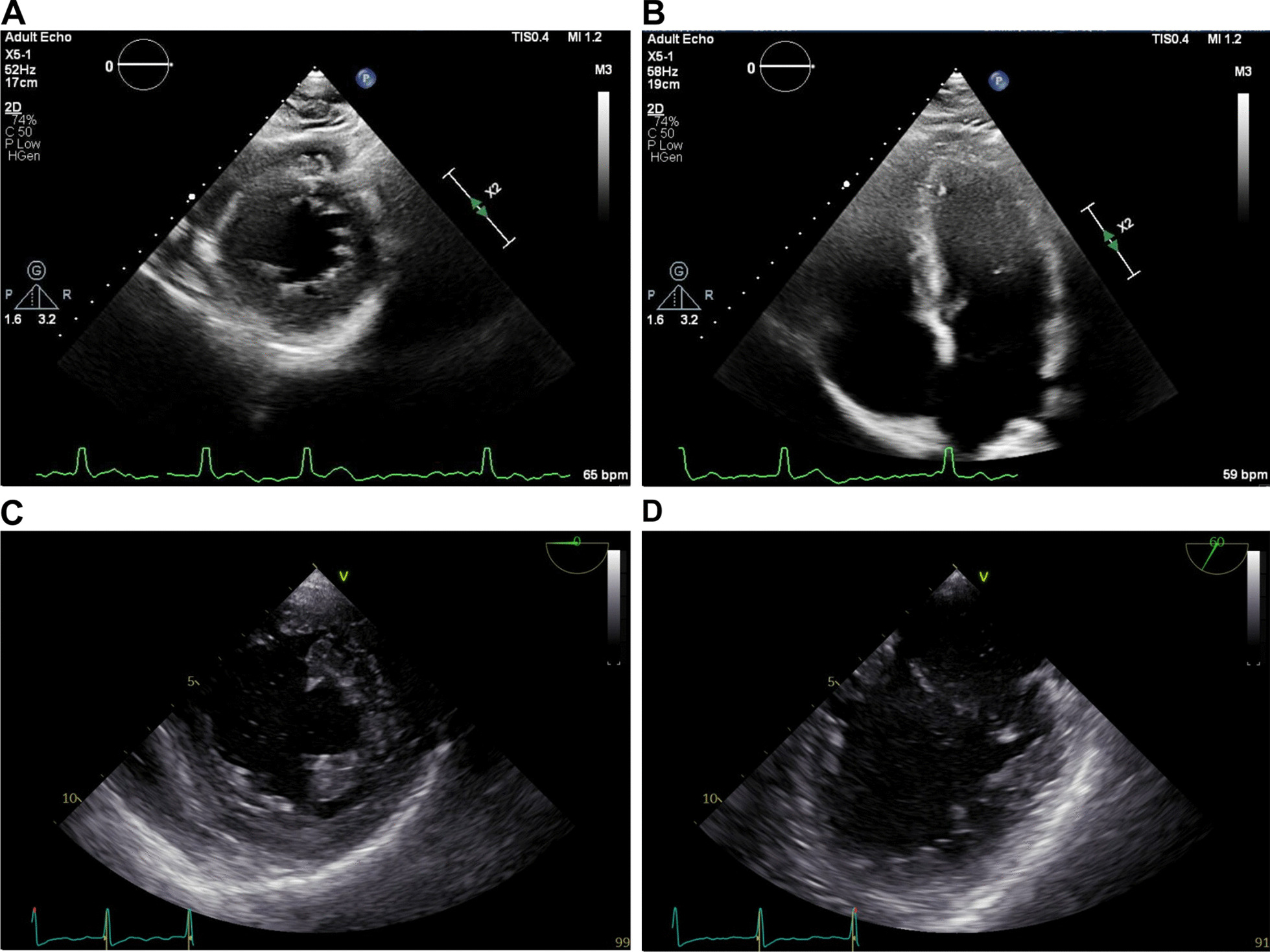
Transthoracic (**A**, **B**) and transesophageal (**C**, **D**) echocardiogram pictures. **A** Parasternal short-axis view; (**B**) apical four chamber view; **C** transgastric midpapillary short-axis view; **D** midesophageal commissural view

Four days after starting gabapentin, the patient presented to the emergency department complaining of palpitations for the past 8 hours. The patient confirmed consumption of one cup of coffee per day and two glasses of wine per week but denied recent alcohol or caffeine binges. The patient also denied family history of Afib. Physical examination showed tachycardia with heart rate of 126 beats per minute with normal blood pressure. Telemetry monitor showed the presence of an irregularly irregular rhythm. Electrocardiogram (EKG) showed atrial fibrillation with rapid ventricular response and left ventricular hypertrophy (Fig. 1A). Laboratory workup including complete blood count with differential, comprehensive metabolic panel, thyroid-stimulating hormone, and toxicology screen, was unremarkable. Transthoracic echocardiogram was performed and showed normal left ventricle (LV) size with mildly increased wall thickness and mild left atrium dilation (volume index of 38 mL/m^2^) (Fig. 2A, B). Gabapentin was discontinued and the patient was started on diltiazem 30 mg per oral four times a day and apixaban 5 mg per oral twice daily. which led to a decrease in the patient’s heart rate to ~60 beats per minute. Transesophageal echocardiogram followed by direct current cardioversion were performed 24 hours after presentation to the emergency department (Fig. 1B). The transesophageal echocardiogram confirmed the presence of mild left atrium dilatation and showed a patent foramen ovale with right-to-left shunt. However, contrary to the previous transesophageal echocardiogram, it showed a normal left ventricle size and wall thickness (Fig. 2C–D). The patient was discharged on diltiazem 120 mg per oral daily and apixaban 5 mg per oral twice daily. Apixaban was stopped 1 month after cardioversion and aspirin 81 mg per oral daily was started.

## Discussion

To the best of our knowledge, this is the first reported young patient with new-onset Afib development associated with gabapentin therapy. Age, high body mass index (> 30 kg/m^2^), height, alcohol consumption (> 1 drink/day), caffeine consumption (> 3 cups/day), hypertension, left ventricular hypertrophy, diabetes mellitus, obstructive sleep apnea, myocardial infarction, heart failure, smoking, and genetic predisposition are well-established risk factors for AFib development (8, 9, 10). In addition, Afib is less prevalent in Asians and African Americans than in individuals with European ancestry (7). Most of these risk factors were validated in cohorts of patients older than 40 years due to the rare prevalence of Afib in young adults. The patient described in the current report had multiple protective factors such as high levels of physical activity (he was training to run a marathon), young age, and his ethnicity. The patient did have left atrial dilatation and ventricular hypertrophy as predisposing factors for development of Afib; however, the degree of atrial dilatation and ventricular hypertrophy were mild at most according to his transthoracic and transesophageal echocardiogram results. A recent meta-analysis showed that the presence of left ventricular hypertrophy carries a relative risk of 1.46 (95% CI 1.32–1.60) for the development of Afib in patients older than 40 years (11). Due to the low incidence of Afib in young, otherwise healthy individuals, this relative risk would translate to a small change in absolute risk. The patient’s left atrial dilatation could be related to the presence of a patent foramen ovale with right-to-left shunt. However, our patient is an athlete and studies confirm that the left atrium is larger in an athletic population compared with nonathletic control groups (12). Meta-analyses describe a 30–37% increase in left atrium volume index in athletes, which could also explain the mild left atrial dilatation seen in this patient (12, 13).

A literature review revealed case reports and case series that describe an association between gabapentinoids and Afib (5, 14, 15, 16, 17). Of these studies, only Ortiz de Landuce *et al.* showed association between gabapentin and Afib, with all the other studies suggesting an association between pregabalin and Afib. The cohort study done by Ortiz de Landuce et al. was restricted to adults aged 65 years or older and only indirectly measured new-onset Afib (defined as initiating an oral anticoagulant and an antiarrhythmic within 3 months). However, it showed that gabapentin was associated with 2.8-fold increased risk of new-onset Afib compared with opiate use and a 2.3-fold increased risk compared with benzodiazepine use. This association was dose dependent, as gabapentin doses ≥ 1200 mg/day were associated with a two-fold higher risk of Afib than lower doses. A more recent cohort study by Harding et al. showed an association between gabapentin and supraventricular ectopy (premature atrial contractions and supraventricular tachycardia) but failed to show an association with Afib (3). Harding et al. used EKG monitoring for the diagnosis of Afib in their study, a sensitive and unbiased method for detecting arrhythmia.

Mechanistically, gabapentin exhibits voltage-gated L-type calcium channel antagonism and attenuates calcium influx by acting on its α2δ subunits, which can explain the increased risk of arrhythmias (2, 3, 5). Therefore, this characteristic may have been the tipping point for a patient with mild left ventricular hypertrophy to develop Afib.

## Conclusions

With rapid increases in gabapentin use, it is important to identify unintended adverse effects of this drug as it is considered a safe alternative to opioids. New-onset atrial fibrillation could be induced by gabapentin in young individuals, therefore gabapentin should be considered as a possible contributing factor once all other possible factors have been excluded.

## Data Availability

All data regarding this case have been reported in this manuscript. Please contact the corresponding author if you are interested in any further information.

## Abbreviations

Afib: Atrial fibrillation
EKG: Electrocardiogram
Kg/m^2^: Kilogram per square meter
km/h: Kilometers per hour
mL/m^2^: Milliliters per square meter
